# Bioactive profiling of *Rumex vesicarius* L. from the Hail region of Saudi Arabia: a study on its phytochemical and elemental analysis, antibiofilm, antibacterial, antioxidant properties, and molecular docking analysis

**DOI:** 10.3389/fmicb.2024.1421143

**Published:** 2024-07-29

**Authors:** Abdel Moneim Elhadi Sulieman, Emad M. Abdallah, Naimah Asid Alanazi, Hajo Idriss, Mohd Adnan, Arshad Jamal, Sohair A. M. Shommo, Mejdi Snoussi

**Affiliations:** ^1^Department of Biology, College of Science, University of Ha’il, Ha’il, Saudi Arabia; ^2^Department of Biology, College of Science, Qassim University, Qassim, Saudi Arabia; ^3^Faculty of Health and Life Sciences, INTI International University, Nilai, Malaysia; ^4^Department of Physics, College of Science, Imam Mohammad Ibn Saud Islamic University, Riyadh, Saudi Arabia; ^5^Deanship of Scientific Research, Imam Mohammad Ibn Saud Islamic University, Riyadh, Saudi Arabia; ^6^College of Education, University of Ha’il, Ha’il, Saudi Arabia

**Keywords:** plants, phytochemical analysis, molecular docking, elemental analysis, antioxidant, antibacterial activity

## Abstract

**Background:**

*Rumex vesicarius* is a wild leafy plant belonging to the family Polygonaceae, renowned for its therapeutic benefits. The genus Rumex comprises approximately 150 species distributed globally.

**Objective:**

The study aimed to investigate the biological activities of *R. vesicarius* using in vitro and *in silico* methods.

**Methods:**

*Rumex vesicarius* was collected from the mountains in Hail and extracted with methanol. The phytochemical composition was qualitatively determined using colorimetric detection methods. Additional analyses included elemental analysis, *in silico* docking, antioxidant, antibacterial, and anti-biofilm properties.

**Results:**

The extract contained various classes of phytochemicals, including flavonoids, phenolics, tannins, terpenes, and saponins. Sixteen constituents were identified through molecular docking, revealing inhibition against the filamentous temperature-sensitive protein Z (FtsZ), a crucial factor in bacterial cell division. Six compounds exhibited low binding scores ranging from −8.3 to −5.0 kcal/mol, indicating efficient interaction at the active site. Elemental analysis identified 15 elements, with potassium being the most abundant, followed by calcium, aluminum, silicon, iron, phosphorus, sulfur, magnesium, titanium, strontium, zinc, manganese, bromine, and chromium. Antioxidant analysis revealed significant properties at lower concentrations compared to ascorbic acid, butylated hydroxytoluene, and β-carotene. Antibacterial analysis demonstrated inhibitory effects on Bacillus subtilis MTCC121 and *Pseudomonas aeruginosa* MTCC 741, with inhibition zones of 13.67 ± 1.0 mm and 11.50 ± 1.0 mm, respectively. The MIC and MBC values ranged from 250 to 500 μg/mL. *R. vesicarius* also exhibited anti-biofilm activity.

**Conclusion:**

Wild-grown *R. vesicarius* from the mountains of Hail is rich in bioactive phytochemicals and essential minerals, exhibiting notable antioxidant and antibacterial properties.

## Introduction

Presently, a diverse array of microbial pathogens represents a pervasive global menace. As per the World Health Organization (WHO), antimicrobial-resistant infections have attained the status of the third-leading cause of mortality on a worldwide scale (primarily bacteria) (Salam et al., 2023). Moreover, studies conducted in 2019 have documented that antimicrobial resistance accounted for approximately 4.95 million fatalities globally. The predominant involvement of multidrug-resistant pathogens, notably Gram-negative bacteria, is evident in most infectious cases (Oliva et al., 2023).

Ironically, microorganisms have evolved ingenious strategies to acquire resistance to drugs, such as forming biofilms. The EPS matrix, known as Extracellular Polymeric Substance, is a physical obstacle that restricts the entry of antibiotics and inhibits the efficient delivery of medications to microbial cells. In addition, microbes that live in biofilms have phenotypic alterations, including decreased rates of growth and modified metabolic pathways, which might make them less vulnerable to antibiotics. In addition, biofilms include persistent cells that are quiescent and physiologically inactive. These cells may avoid the effects of antibiotics and function as reservoirs for recurring infections (Rather et al., 2021a,b). Medicinal plants may serve as crucial agents in preventing the production of biofilms, as they utilize mechanisms distinct from antibiotics (Al-Mijalli et al., 2023a). Green nanotechnology has attracted considerable attention in the development of modern pharmaceuticals. Therefore, nanomaterials (NMs) have been considered “a wonder of contemporary medicine” and are promoted as powerful agents for fighting against biofilms (Rather et al., 2022; Rather and Mandal, 2023).

With an increasing global emphasis on health consciousness, there is a rising demand for herbal medicines due to their multiple advantages, including being safer, more affordable, and having fewer side effects than artificial modern drugs (Zhao et al., 2023). Medicinal plants are experiencing substantial demand in worldwide markets. As per the Food and Agriculture Organization (FAO), the international trade of herbal plants exhibited significant growth, escalating from 1.3 billion USD in 1996 to 120 billion USD in 2019 (Bhatkar and Shirkole, 2023). Considering that approximately 80% of the world’s population relies on medicinal plants for their primary healthcare, a promising future is seen for maintaining health using medicinal plants (Riasat et al., 2023).

*Rumex vesicarius* L. (*R. vesicarius*) is a member of the Polygonaceae family. This family contains 50 genera and approximately 1200 species of flowering weeds. Noteworthy among these are the genus Eriogonum, with 2410 species; *Rumex*, with 200 species; Cocoloba, with 120 species; and Persicaria, with 100 species, among others (Tonny et al., 2017). *R. vesicarius* is a consumable green leafy plant. Indigenous to the desert and semi-desert areas in Northern Africa and Southwest Asia. This weed exhibits an annual growth pattern, thriving particularly during the fall and spring rainy seasons (Beddou et al., 2015). In traditional medicine, *R. vesicarius* is employed by traditional healers to address a range of health concerns, including hepatic diseases, constipation, heart issues, indigestion, pain, spleen disorders, flatulence, dyspepsia, asthma, bronchitis, vomiting, toothache, piles, scabies, and leucoderma, as well as for its claimed properties as an appetizer, analgesic diuretic, stomachic, tonic, and laxative (El-Hawary et al., 2011; Mostafa et al., 2011; Salama et al., 2022).

The Hail region is in the northern, central part of Saudi Arabia. The natural plant life in the Hail area covers a significant expanse, coexisting with a diverse array of animals and plants. It is the home of various plant species, which Bedouins have used for multifaceted purposes, including as a source of food, herbal medicine, and nutrition for grazing animals (Sulieman et al., 2023a,b).

Local people use this plant for its many uses, including as an appetite stimulant, diuretic, astringent, remedy for snake and scorpion bites, and diarrhea; they consume roasted seeds as an antidote. Therefore, the current study aimed to explore the biological activities of this plant by investigating the chemical constituents of *R. vesicarius* grown wildly in harsh conditions of the mountainous area of Hail province, evaluating its antimicrobial properties, assessing antioxidant activity, and conducting docking analysis to predict molecular interactions.

## Materials and methods

### Sample collection and the study area

**FIGURE 1:**
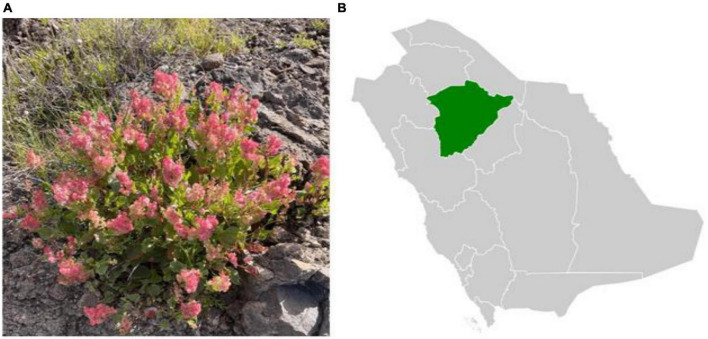
Map of Saudi Arabia showing the collection area of *Rumex vesicarius*. **(A)** *Rumex vesicarius*, **(B)** Location of Hail region in green.

The botanical specimens of *R. vesicarius*, locally referred to as “Humaid,” were systematically gathered from Aja Mountain in the Hail region of Saudi Arabia during the spring season, spanning from March to May 2022, with temperature ranging from 27.9 to 33.2°C. We collected aerial parts from at least five individuals of the same plant. The collection site’s geographic coordinates are from 41°45′ E to 43°00′ E longitudes and from 27°25′ N to 30°00′ N latitudes. The study area, situated in the northern middle part of Saudi Arabia, encompasses an extensive expanse of 118,322 square kilometers, equivalent to 6% of the entire landmass of Saudi Arabia. This region contains an exposed complex of Precambrian igneous and metamorphic rocks, which form an integral part of the extensive Phanerozoic formations, overlaying the northern and eastern peripheries of the Arabian Shield. Aja Mountain, positioned between Hail town and the An-Nafud desert, is the largest mountain in this region. *R. vesicarius* is used by some Bedouins in the Hail region to treat digestive problems (personal communication). The vegetation of these mountains is notably influenced by the flora of Mediterranean countries, contributing to a distinctive ecological landscape. Most of the Hail region’s plant species are annual (Figure 1).

### Chemicals

All the chemicals, including methanol, ethanol, and chloroform, employed in the study were of analytical grade. These chemicals and indicators were in their original state without any purification processes. They procured from Merck (Merck^®^, USA). Folin–Ciocalteu’s phenol reagent (ACS reagent grade), aluminum chloride hexahydrate (reagent grade), quercetin [≥ 95% (HPLC), solid], H_2_SO_4_ (analytical grade ≥ 95%). Standard vitamin E (USP grade) and DPPH (2,2-Diphenyl-1-picrylhydrazyl) reagents (ACS reagent grade) were obtained from Sigma-Aldrich (St. Louis, MI, USA). Bacterial media were bought from Oxoid, UK.

### Bacterial strains

Bacterial strains, namely *B. subtilis* (MTCC121) and *P. aeruginosa* (MTCC741), were obtained from the Microbial Type Culture Collection (MTCC) in Chandigarh, India, and cultured in our laboratory on Muller-Hinton agar. A singular bacterial colony was then transferred to fresh media and allowed to incubate overnight at 37°C, facilitating the development of bacterial cultures. The turbidity of the culture was adjusted to confirm the 0.5 McFarland standard (10^6^ CFU/mL) using a sterile typical saline solution (0.9%).

### Preparation of plant extract

Fresh samples of *R. vesicarius* aerial parts (whole plant) were collected from the mountains and identified by the Department of Biology, College of Science, University of Ha’il, Saudi Arabia. A specimen (voucher specimen no. HAU002021/2) was deposited. We dried the obtained *R. vesicarius* in the shade for up to a week. Subsequently, we ground the entire plant material using a mechanical grinder (Electric Grinder, OMCG2145, Olsenmark, New York, NY, USA) to achieve a finer particle size, optimizing solvent extraction efficiency. 100 g of the plant’s powder was macerated in 1,000 mL of 80% methanol for up to three days in darkness at room temperature (approximately 35–37°C) with regular agitation. Previous studies have shown that methanol is the optimal solvent for extracting plant compounds, as it yields the highest extraction efficiency, particularly with an 80% methanol solution (Anwar and Przybylski, 2012; Razali et al., 2012). The methanol was then evaporated, and the resulting extract was concentrated using a rotary vacuum evaporator (8 kW, 50 L, Henan Lanphan Industry Co., Ltd., Zhengzhou, China) (Sulieman et al., 2023a). Furthermore, the unfiltered extracts were dissolved in hexane and micro-filtered before injected into the GC-MS.

### Qualitative phytochemical profile

The determination of major phytochemical compounds in the crude methanol extract was conducted using standard qualitative methods to detect saponins, terpenes, flavonoids, phenols, tannins, alkaloids, and cardiac glycosides, as reported by Banso and Adeyemo (2006) and Raaman (2006).

### Quantitative tests for total phenolic, flavonoid and tannin

#### Determination of total phenolic content

The total phenolic content (TPC) of various extracts was measured using the Folin-Ciocalteu reagent, following the procedure outlined by Kumar et al. (2013). The samples were examined at a concentration of 1 mg/mL. Subsequently, the extract underwent a ten-fold dilution with deionized water and was placed in a test tube with 0.75 mL of Folin-Ciocalteu reagent, where it was stirred. The resultant mixture was allowed to stand at 25°C for 5 min. The liquid was gently mixed after adding 0.75 mL of a saturated sodium carbonate solution. The absorbance at 725 nm was then measured after 90 min at 25°C using a UV-Vis spectrophotometer. A calibration curve was developed using gallic acid, and the total phenolic content was quantified in terms of gallic acid equivalents (GAE) in mg of gallic acid/mg of dry weight.

#### Determination of total flavonoid content

The extracts’ total flavonoid concentration (TFC) was quantitatively determined following a previously published methodology with minor modifications (Zhao et al., 2018). A volume of 1.5 mL (1 mg/mL) of the extract was combined with an equal amount of 2% AlCl_3_–6H_2_O. After 10 min of incubation, the mixture underwent vigorous agitation, and the absorbance at 367 nm was recorded. A quercetin calibration line measured the total flavonoid content in milligrams of quercetin (mg QE) per gram of dry weight (mg QE/g). Each sample underwent three tests.

#### Determination of total tannin content

Tannins were quantified using a colorimetric method involving a modified vanillin test (Selvi et al., 2012). A volume of 1.5 mL of concentrated H_2_SO_4_ and 3 mL of a 4% methanolic vanillin solution was added to 50 mL of the extract (1 mg/mL). After allowing the mixture to stand for 15 min, the absorbance at 500 nm was measured using methanol or water as a reference. Total tannin content was determined as mg catechin/g of dry weight or mg CE/mg. Each sample underwent three replication analyses.

### The DPPH assay

DPPH of extracts and standard vitamin E were assessed using Chakraborty and Paulraj’s (2010) technique. Different concentrations of extracts (20 mg/mL) and standard (1 mg/mL) were pipetted into tiny tubes. A 0.5 mL amount of DPPH∙ methanolic solution was added to each sample and standard. For 30 min, the blend was left at 25°C in the dark. The solution’s absorbance was measured at 520 nm using a spectrophotometer. 0.5 mL of DPPH solution and 0.5 mL of methanol were mixed for the control.

The blank was pure methanol. The DPPH inhibition (PI%) is determined using the equation (Equation 1): PI (%) = 100 x (A_*control*_—A_*sample*_) /A_*control*_, where A_*control*_ and A_*sample*_ are the absorbances of the control solution and test sample or standard, respectively.

### ABTS radical scavenging assay

ABTS cation scavenging activity test, also known as 2,2′-casino-bis (3-ethylbenzthiazoline-6-sulphonic acid), was employed for an antiradical assay (Chakraborty and Paulraj, 2010). Radical monocation was achieved by adding 2.45 mM K_2_S_2_O_8_ to ABTS solution (7 mM). The combination was left at room temperature for 15 h in the dark. Samples were dissolved in methanol and distilled water. Variable extract quantities and tocopherol (vitamin E). The test was conducted and compared to the standard. The antioxidant activity was evaluated by mixing 800 mL of diluted ABTS+ with 200 mL of each standard and sample. After 30 min, we measured absorbance at 734 nm using spectrophotometry. Each measurement was tripled. Percent (%) inhibition was utilized to reflect the antioxidant capacity of test samples and reference material.

The % scavenging of ABTS+ radicals was estimated using Equation 2:


PI(%)=100×(A-c⁢o⁢n⁢t⁢r⁢o⁢lA)s⁢a⁢m⁢p⁢l⁢e/A,c⁢o⁢n⁢t⁢r⁢o⁢l

A_*control*_ and A_*sample*_ represent the absorbance of the control and test sample or standard, respectively.

### Gas Chromatography-Mass Spectrometry (GC-MS) analysis

**FIGURE 2:**
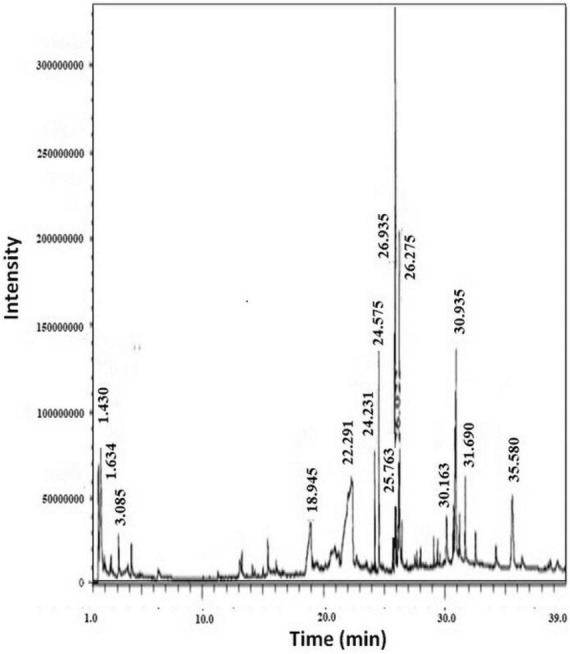
GC-MS chromatogram of *R. vesicarius* crude extract.

The GC-MS from Perkin Elmer Clarus 600 was utilized to analyze the crude extract of *R. vesicarius*. The GC-MS consisted of a Rtx 5MS capillary column (30 m length, 0.25 mm inner diameter, and 0.25 m film thickness). The system was capable of reaching a maximum temperature of 350°C. Furthermore, the GC system was coupled to a Perkin Elmer Clarus 600C MS. The carrier gas employed in the present investigation was ultra-high-quality helium (99.9999%), maintained at a constant 1.0 mL/min flow rate. The operational temperatures of the ion source, transfer line, and injector were recorded as 280°C, 270°C, and 270°C, respectively. We measured the ionizing energy of the system to be 70 electron volts (eV). The electron multiplier (EM) voltage was acquired by using auto-tune. We collected the data by conducting comprehensive full-scan mass spectrometry within 40 to 550 atomic mass units (AMU). The compound fragmentation patterns from the mass spectra were compared to those stored in the spectrometer database utilizing the National Institute of Standards and Technology’s (NIST) Mass Spectral Library (Lalitha et al., 2021). Table 1 and Figure 2 show the experimental parameters employed for the GC-MS system analysis.

**TABLE 1:** The GC-MS analysis conditions.

Parameter	Condition
Column oven temperature	70.0°C
Injection temperature	250.00°C
Injection mode	Split
Flow control mode	Column flow
Pressure	61.3 kPa
Total flow	24.0 mL/min
Column flow	1.00 mL/min
Purge flow	36.7 cm/sec
Split ratio	3.0 mL/min
Time	40.00 min

### Molecular docking

Molecular docking was performed using AutoDock Vina software (Ver. 1.2.5) (Löwe and Amos, 1999), targeting the filamentous temperature-sensitive Z (FtsZ). The 3D crystal structure for FtsZ was obtained from the RCSB protein data bank website (PDB ID: 1OFU). The structure of the ligands and PC190723 was obtained from the PubChem database. Regarding the non-availability of 3D, the obtained 2D was converted to 3D via the OpenBabel online converter (Kusuma et al., 2019). Their energies were minimized using UCSF Chimera, which was used with the Discovery Studio visualizer for imaging and viewing ligand-protein interactions (Vanjare et al., 2023).

### Element analysis

The elemental quantities of the *R. vesicarius* sample were determined by energy dispersive X-ray fluorescence (EDXRF) analysis conducted by Thermo Fisher Scientific^®^ (USA). The device is built with a cutting-edge silicon drift detector (SDD) that effectively removes spectrum interference and provides a quick response. The 30 mm^2^ active area facilitates a broad hard angle, enhancing X-ray collection efficiency. The high-flux rhodium anode tube has been specifically engineered to enable direct excitation of X-ray tubes or customized excitation through various filters, thereby enhancing the sensitivity to different elements (Shaltout and Hassan, 2021).

### Antioxidant activity

#### DPPH radical scavenging method

The free radical scavenging activity of the extracts was assessed using the DPPH radical scavenging method, with vitamin E as the standard (Sulieman et al., 2023b). The stock solutions and standard concentration were 20 mg/mL and 1 mg/mL, respectively. Each sample and the standard were combined with an equal volume (0.5 mL) of DPPH methanolic solution. Following vigorous stirring, the mixture was allowed to stand for 30 min at a temperature of 25°C in the dark. The absorbance of the resulting solution at 520 nm was measured using a spectrophotometer, and measurement was performed thrice. The control solution was prepared by combining 0.5 mL of the DPPH solution with 0.5 mL of methanol, while pure methanol served as the blank. The percentage (PI%) of free radical DPPH inhibition was calculated using the equation:


$$PI\%\;=\;100\;\times \;\left(A_{Control}-A_{Sample}\right)/A_{Control}$$

Where, *A*_*Control*_ and *A*_*Sample*_ are the absorbance of the control solution and a test sample or standard.

#### ABTS radical scavenging activity method

The antiradical assay was conducted utilizing the ABTS cation scavenging activity test involving 2,20-casino-bis (3-ethylbenzthiazoline-6-sulphonic acid), commonly known as ABTS (Chakraborty and Paulraj, 2010). The ABTS radical mono-cation was generated by reacting to a 7 mM ABTS solution with 2.45 mM potassium persulfate (K_2_S_2_O_8_), allowing the combination to stand at room temperature and in the dark for 15 h. Samples of the organic extracts were dissolved in methanol. The tocopherol (vitamin E) standard and the extract concentration were evaluated against the benchmark. The antioxidant activity was determined by mixing 800 mL of diluted ABTS with 200 mL of each standard and sample, and the absorbance was measured spectrophotometrically at 734 nm after 30 min. Each measurement was conducted three times. The antioxidant capacity of the test sample and the standard was expressed as a percentage (%) of inhibition. The proportion of ABTS scavenging was calculated using the following equation:


$$PI\%\;=\;100\;\times \;\left(A_{Control}-A_{Sample}\right)/A_{Control}$$

Where, *A*_*Control*_ and *A*_*Sample*_ are the absorbance of the control solution and a test sample or standard.

#### β-carotene/linoleic acid method

The extracts’ ability to inhibit the bleaching of β-carotene was evaluated using a previously described method (Ikram et al., 2009). When linoleic acid is heated with β-carotene and linoleic acid compounds, it produces a free radical. 20 mL of linoleic acid and 200 mL of Tween-20 were combined with 2 mL of the β-carotene solution (1.5 mg β-carotene/2.5 mL chloroform). Using a rotary evaporator, the chloroform was evaporated at 40°C under vacuum. The resulting dried material was then mixed with 50 mL of distilled water to form a β-carotene-linoleic acid emulsion. The capacity of each extract to bleach β-carotene was examined by adding 0.800 mL of the emulsion to 0.200 mL of extracts at varied concentrations (stock solution 20 mg/mL). The absorbance at 470 nm was measured before and after the combinations were incubated for 120 min at 50°C in a water bath. Each test was performed three times. The antioxidant activity of the extracts was calculated using the following formula:


PI%=1-(A-0A/tAc-0Ac)t×100

Where, the absorbance values of the test sample, standard, or control recorded at zero time are represented by A_0_ and Ac_0_, respectively. In contrast, the corresponding absorbance values measured after 120 min are denoted by A_*t*_ and Ac_*t*_ at zero time, respectively, and A_*t*_ and Ac_*t*_ are the corresponding absorbance values measured after incubation for 120 min, respectively.

### Antibacterial activity

#### Disk-diffusion test

Following a previously published method, we used the disk-diffusion test to assess the antibacterial efficacy of the methanolic extract from *R. vesicarius* (Sulieman et al., 2023a). In brief, suspensions of the tested bacteria, *B. subtilis* MTCC121 and *P. aeruginosa* MTCC741, brought from our microbiology lab in broth culture, and the bacteria were sub-cultured on Nutrient agar (Sigma Aldrich^®^, USA), and routinely examined using Gram staining and appropriate biochemical tests. Then, we adjusted bacteria to McFarland turbidity to attain approximately 10^6^ CFU/mL. These suspensions were then transferred onto sterile plates containing 20 mL of Mueller-Hinton agar. Under aseptic conditions, two sterile filter disks with a diameter of 6 mm (Whatman No. 1) were placed onto agar plates previously inoculated with bacteria after being saturated with 1,000 μg/ml of the adjusted methanolic crude extract (the 6 mm disk absorbs 10 μl). Disks solely impregnated with 80% methanol were employed as a negative control, while ampicillin, at 10 μg/disk, served as a positive control (standard drug). The plates were incubated at room temperature for 24 h. Subsequently, we calculated the mean value based on three individual repetitions.

#### Determination of MIC and MBC

The microdilution method was employed on 96-well microplates to estimate the methanolic extract’s minimum inhibitory concentration (MIC) from *R. vesicarius*. In summary, serial two-fold dilutions of the extract were prepared, ranging from 500 to 31.25 μg/mL, using 5% DMSO for each row of microplates. Subsequently, the microplates were incubated with 20 μL of bacterial suspensions adjusted to 0.5 McFarland and 160 μL of Mueller-Hinton broth for 24 h at 37°C. We assessed the bacterial growth by incubating 40 μL of 2,3,5-triphenyl tetrazolium chloride (TTC) at a 0.2 g/mL concentration for 30 min at 37°C. TTC identified wells with bacterial growth by staining bacterial cells with a red dye. The MIC was determined in the microplate wells with the least amount of extract and no discernible bacterial growth. On the other side, the minimum bactericidal concentration (MBC) of the methanol extract from *R. vesicarius* against the tested bacteria was determined using the agar diffusion test. Mueller-Hinton agar plates were inoculated with 50 μL from the tubes of the MIC test that exhibited no apparent growth with *B. subtilis* and *P. aeruginosa*. These plates were incubated for up to 24 h at 35–37°C, and we examined the bacterial growth after incubation. The lowest concentration preventing the growth of even a single bacterial colony on the Mueller-Hinton agar plate was defined as the MBC value. The MBC/MIC ratio was also calculated (Al-Mijalli et al., 2023b).

#### Antibiofilm method

The impact of the methanol extract from *R. vesicarius* on the formation of biofilms by *B. subtilis* and *P. aeruginosa* was evaluated through the crystal violet staining assay (Venkatramanan et al., 2020). The assay used 96-well microplates in Mueller-Hinton broth supplemented with 1% glucose. Sub-inhibitory concentrations (sub-MIC) of *R. vesicarius* extract (1/2 MIC and 1/4 MIC) were introduced, with bacterial strains adjusted to approximately 10^6^ cells/mL loaded into the wells. Positive controls included Mueller-Hinton broth and 80% methanol (as the solvent for the crude extract) without the extract. Incubation occurred at 30°C for 24 h in the 96-well microplates. After incubation, planktonic cells were meticulously aspirated and removed from the wells, followed by three washes with phosphate-buffered saline (PBS, pH 7.4). The microplate was air-dried for 24 h, and the wells containing the formed biofilms were stained with a 0.3% (w/v) crystal violet solution (200 μL) for 15 min at room temperature. After staining, wells were subjected to three PBS washes to eliminate unabsorbed dye. Subsequently, glacial acetic acid (30% v/v) was employed to dissolve crystal violet, and the microplate was read at 570 nm. The percentage of biofilm inhibition was calculated using the provided equation:


$$B\,i\,o\,f\,i\,l\,m\,i\,n\,h\,i\,b\,i\,t\,i\,o\,n\%=\frac{O\,D\,\left(c\,o\,n\,t\,r\,o\,l\right)-O\,D\,\left(t\,e\,s\,t\right)}{O\,D\,\left(c\,o\,n\,t\,r\,o\,l\right)}\times 100$$

### Statistical analysis

Data analysis was performed using the SPSS application for Windows, version 20 (SPSS Inc., Chicago, IL, USA). Differences between groups were determined through a one-way ANOVA, and further insights were obtained using Duncan’s multiple-range test. The results were presented as means and standard error of means (Std. Err.) or standard deviation, with statistical significance set at *p* ≤ 0.05.

## Results

### Phytochemical properties of *R. vesicarius*

The plant extract of *R. vesicarius* exhibited notable positive phytochemical outcomes, as evidenced by significant color changes observed through qualitative assessment, as shown in Table 2. The prevalent chemicals identified in the plant following the screening included flavonoids, phenolics, tannins, terpenes, and saponins. Figure 2 displays the GC-MS chromatogram of the methanol extract obtained from *R. vesicarius* crude extract. The chromatograms were analyzed by comparing various parameters, such as peak retention duration, peak area (%), height percentage, and mass spectrum fragmentation characteristics, with the corresponding values for standard substances available in the National Institute of Standards and Technology (NIST) library. GC-MS analysis of the extract identified sixteen chemical compounds. Table 3 displays the chemical compounds’ respective percentages in the *R. vesicarius* crude extract. The identified chemical compound concentrations were in the order of 6,9,12,15-Docosatetraenoic acid, methyl ester (17.63%) > Glycidyl isobutyl ether (11.35%)> Dichloroacetic acid, tridec-2-ynyl ester (10.31) > Rockogenin (9.28) > 9,12-Octadecadienoic acid (Z,Z)-, methyl ester (7.15%) > n-Hexadecanoic acid (6.62%) > Glyceryl linolenate (6.37) > Isoamyl nitrite (6.12%) > S-Methyl methanethiosulphonate (3.97) > D-Amygdalin (3.87) > Phytol (3.62%) > 13-Docosenamide, (Z)-(3.57%) > Methyl stearidonate (Methyl isohexadecanoate) (3.45%) > Glyceraldehyde (3.33%) > Alpha.-d-Mannofuranoside, isopropyl- (1.34%).

**TABLE 2:** Phytochemical profile of *R. vesicarius* plant extract.

Plant extract	Saponins	Terpenes	Flavonoids	Phenols	Tannins	Alkaloids (Dragendorff)	Alkaloids (Mayer)	Cardiac glycosides
*R. vesicarius*	+	+	+	+	+	−	−	−

+: present, −: absent.

**TABLE 3:** Major compounds were identified via GC-MS analysis from the crude extract of *R. vesicarius*.

No.	Compounds	Area%	RT (min.)	Formula	MW (g/mol)	Structure
1	S-Methyl methanethiosulphonate	3.97	1.430	C_2_H_6_O_2_S_2_	126.20	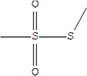
2	Isoamyl nitrite	6.12	1.634	C_5_H_11_NO_2_	117.15	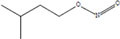
3	Glyceraldehyde	2.02	3.085	C_3_H_6_O_3_	90.08	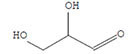
4	Glycidyl isobutyl ether	11.35	18.945	C_7_H_14_O_2_	130.18	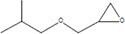
5	Alpha.-d-Mannofuranoside, isopropyl-	1.34	22.291	C_9_H_18_O_6_	222.24	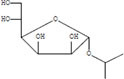
6	Methyl isohexadecanoate	3.33	24.231	C_17_H_34_O_2_	270.5	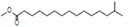
7	n-Hexadecanoic acid	6.62	24.575	C_16_H_32_O_2_	256.42	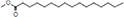
8	Methyl stearidonic	3.45	25.763	C_19_H_30_O_2_	290.4	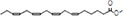
9	Linoleic acid methyl ester	7.15	25.874	C_19_H_34_O_2_	294.5	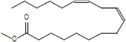
10	6,9,12,15-Docosatetraenoic acid, methyl ester	17.63	25.935	C_23_H_38_O_2_	346.5	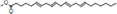
11	Phytol	3.62	26.022	C_20_H_40_O	296.5	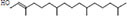
12	Dichloroacetic acid, trident-2-vinyl ester	10.31	26.275	C_15_H_24_C_l2_O_2_	307.3 g	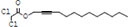
13	D-Amygdalin	3.87	30.163	C_20_H_27_NO_11_	457.4	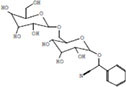
14	Glyceryl linoleate	6.37	30.935	C_21_H_36_O_4_	352.5	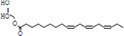
15	Erucylamide	3.57	31.69	C_22_H_43_NO	337.6	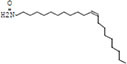
16	Rockogenin	9.28	35.58	C_27_H_44_O_4_	432.6	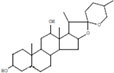

RT, retention time, MW, molecular weight.

### Docking analysis of compounds from *R. vesicarius*

**FIGURE 3:**
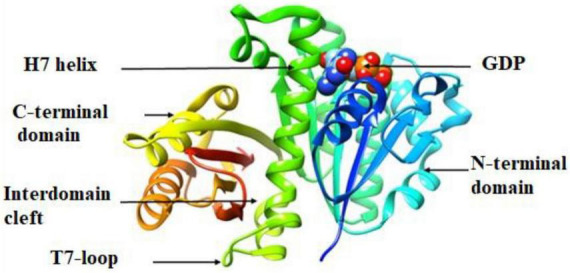
Crystal structure of *P. aeruginosa* FtsZ (Protein Data Bank ID code 1OFU).

**FIGURE 4:**
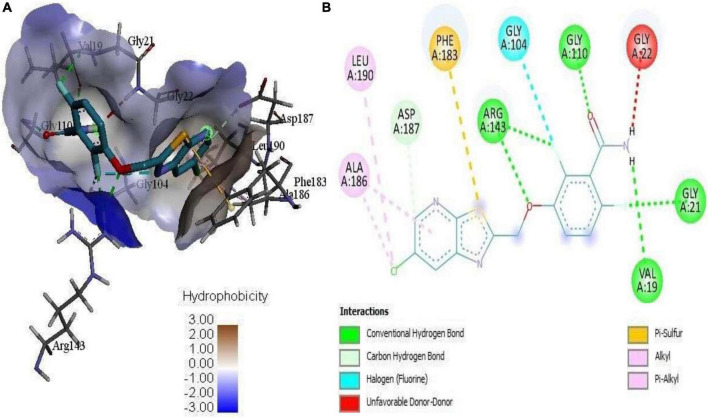
**(A)** 3D complex form of the reference PC190723 with FtsZ docked to the GPT site pocket and **(B)** bond interactions in 2D.

**FIGURE 5:**
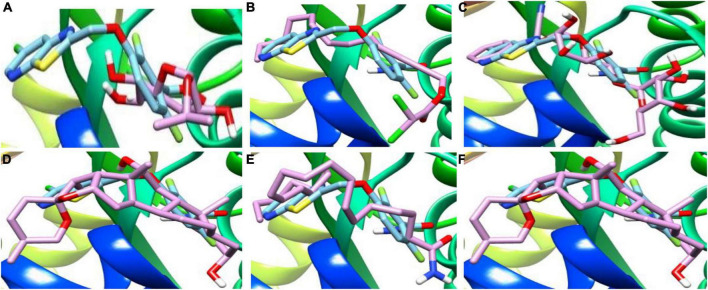
3D complex of the reference PC190723 (with hydrocarbon cyan) at FtsZ GPT. site superimposed with **(A)** Alpha-d-Mannofuranoside, isopropyl-, **(B)** Dichloroacetic acid, tridec-2-ynyl ester, **(C)** D-Amygdalin, **(D)** Glyceryl linolenate, **(E)** Erucylamide, **(F)** Rockogenin.

**FIGURE 6:**
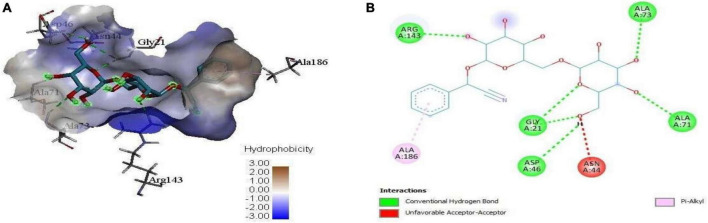
**(A)** 3D complex form of D-Amygdalin with FtsZ docked to the GPT site pocket and **(B)** bond interactions in 2D.

The molecular docking results (*in silico*) are represented in Figures 3–6 and Table 4. We have detected sixteen constituents, and molecular docking analysis unveiled their inhibitory potential against the filamentous temperature-sensitive protein Z (FtsZ), a critical regulator in bacterial cell division. Notably, six of these compounds demonstrated notable efficiency in interacting with the active site, as evidenced by their low binding scores falling within the range of −8.3 to −5.0 kcal/mol. The filamentous temperature-sensitive Z (FtsZ) is a cytoplasmic guanosine triphosphate (GTP) protein (Figure 3). It is highly conserved in most prokaryotic organisms and is a homolog of the eukaryotic cytoskeletal tubulin. GTP binding and hydrolysis are involved in FtsZ polymerizing into the Z-ring form, which divides the mother cell into two new cells.

**TABLE 4:** The binding affinity of the identified via GC-MS analysis from the crude extract of *R. vesicarius* with FtsZ protein.

Compound ID	Score energy (Kcal/mol)	RMSD	Binding percent (%)	H bond interaction (Conformers No., Interacted bonds), (range D-H.A dist), (residues)
PC190723	−7.9 to −6.3	0.0 to 7.8	78	(7/23), (1.76 Å to 3.05 Å) (GLY 21, GLY 22, ASN 44, GLY 108, THR 109, GLY 110, ARG 143, VAL 19, THR 103, GLU 139, ASP 187)
Alpha.-d-Mannofuranoside, isopropyl-	−5.4 to −5.1	0.0 to 8.0	33	(3/11), (1.89 Å to 2.48 Å) (GLY 22, ASN 44, GLY 104, THR 109, GLY 110, ARG 143, VAL 19, THR 133)
Dichloroacetic acid, tridec-2-vinyl ester	−5.0	0.0 to 0.0	11	(1/2), (2.12 Å to 2.22 Å) (GLY 21, ASN 44)
D-Amygdalin	−8.3 to −7.1	0.0 to 4.5	100	(9/40), (1.84 Å to 2.67 Å) (GLY 21, ASN 44, ALA 71, GLY 108, THR 109, GLY 110, ARG 143, ASP 46, GLY 104)
Alpha.-Glyceryl linoleate	−5.8 to −5.0	0.0 to 9.2	67	(6/17), (1.93 Å to 2.63 Å) (GLY 21, GLY 22, GLY 23, ASN 44, ALA 71, GLY 104, GLY 108, THR 109, GLY 110, THR 133)
Erucylamide	−5.0	0.0 to 0.0	11	(1/1), (2.17 Å) (MET 105)
Rockogenin	−7.1	18.0 to 23.2	11	(1/2), (2.04 Å to 2.25 Å) (GLY 21, ASN 44)

RMSD, the root mean square deviation.

### Element contents of *R. vesicarius*

In the current study, the aerial parts of *R. vesicarius* underwent quantitative elemental analysis. As shown in Table 5, the elemental analysis of *R. vesicarius* resulted in the detection of 15 elements. From the table, it can be noticed that the mineral concentrations were in the order of K > Ca > Cl > Al > Si > Fe > P > S > Mg > Ti > Sr > Zn > Mn > Br > Cr. The elements present in the highest quantities were K (29.02 ± 0.20 m/m%), Ca (21.13 ± 0.16 m/m%), and Cl (10.62 ± 0.15 m/m%). In contrast, Si, Fe, P, S, and Mg were found in relatively lower amounts, ranging from 4.14 ± 0.10 m/m% to 0.89 ± 0.12 m/m%. The trace elements, namely Ti, Sr, Zn, Mn, Br, and Cr, were detected in minimal quantities, ranging from 0.260 ± 0.013 to 0.0159 ± 0.0057 m/m%. Mineral elements play distinct and indispensable roles in plant metabolism, categorized into macronutrient elements (N, P, S, K, Mg, Ca), micronutrient elements (Fe, Mn, Zn, Cu, B, Mo, Cl, Ni), and beneficial elements (Na, Si, Co, Al)^37^. Al, Co, Na, Se, and Si are beneficial plant elements.

**TABLE 5:** Mineral composition of *R. vesicarius* (aereal parts) using energy dispersive X-ray fluorescence analysis.

Compound	Concentration (Mass Percent, m/m%)	Std. Err.	Element	Concentration (mass percent, m/m%)	Std. Err.
K_2_O	34.96	0.24	K	29.02	0.20
CaO	29.55	0.23	Ca	21.13	0.16
Cl	10.62	0.15	Cl	10.62	0.15
Al_2_O_3_	7.83	0.19	Al	4.14	0.10
SiO2	6.07	0.14	Si	2.84	0.07
Fe_2_O_3_	3.88	0.10	Fe	2.72	0.07
P_2_O_5_	2.45	0.17	Px	1.07	0.07
SO_3_	2.39	0.20	Sx	0.957	0.080
MgO	1.48	0.20	Mg	0.89	0.12
TiO_2_	0.434	0.022	Ti	0.260	0.013
SrO	0.110	0.005	Sr	0.0933	0.0046
ZnO	0.0875	0.0044	Zn	0.0703	0.0035
MnO	0.0610	0.0081	Mn	0.0472	0.0063
Br	0.0311	0.0015	Br	0.0311	0.0015
Cr_2_O_3_	0.0232	0.0084	Cr	0.0159	0.0057

Std. Err., standard error.

### Antioxidant properties of *R. vesicarius*

Table 6 shows the evaluation results of *R. vesicarius*’s antioxidant properties, which included measurements of total tannins, flavonoids, DPPH scavenging activity, β-carotene, and ABTS IC_50_ (mg/mL). The results show that the compounds have antioxidant activities, even at lower concentrations than the typical molecules like β-carotene, butylated hydroxytoluene (BHT), and ascorbic acid (AA). Analyzing its antioxidant properties revealed that it was more effective than ascorbic acid, butylated hydroxytoluene, and β-carotene at lower doses (Table 6). The total tannins, total flavonoids, and total phenols in *R. vesicarius* were measured at 28.08 + 0.20 mg TAE/g, 153.58 + 0.87 mg QE/g, and 14.56 + 0.29 mg GAE/g, respectively. Examining how an antioxidant affected the DPPH free radical’s stability was the study’s primary goal. Finding the sample concentrations that inhibited DPPH radical scavenging activity by 50% (IC_50_) was the specific goal of the investigation. With an average IC_50_ value of 0.08 + 0.002 mg/mL, the *R. vesicarius* methanol extract showed vigorous antioxidant activity.

**TABLE 6:** Antioxidant activities of *R. vesicarius* methanol extract (80% v/v) compared to known drugs.

The experiment	*R. vesicarius* Extract	Butylated hydroxytoluene	Ascorbic Acid
**Phytochemical screening**			
Total Phénols (mg GAE/g extract)	14.56 ± 0.29	–	–
Total Tannins (mg TAE/g extract)	28.08 ± 0.20	–	–
Total Flavonoïdes (mg QE/g extract)	153.58 ± 0.87	–	–
**Antioxidant assays**			
DPPH IC_50_ (mg/mL)	0.08 ± 0.002	0.023 ± 0.34	0.022 ± 0.54
ABTS IC_50_ (mg/mL)	0.152 ± 0.003	0.018 ± 0.044	0.021 ± 0.001
β-carotene IC_50_ (mg/mL)	0.42 ± 0.02	0.042 ± 0.35	0.017 ± 0.001

### Antibacterial properties of *R. vesicarius*

We evaluated the overall antibacterial efficacy of the *R. vesicarius* extract through a disk diffusion assay, and the results are presented in Table 7. The data in the table indicates that the highest susceptibility to the *R. vesicarius* extract was exhibited by *B. subtilis*, as evidenced by the most expansive mean zone of inhibition measuring 13.67 ± 1.5 mm, followed by *P. aeruginosa*, with a zone of inhibition measuring 11.50 ± 1.0 mm. The findings were consistent with the positive control tested (ampicillin 10 μg/disk) and statistically significant at *p* ≤ 0.05. The Gram-positive bacterium (*B. subtilis*) generally exhibited heightened susceptibility compared to the Gram-negative bacterium (*P. aeruginosa*). The MIC assay was carried out to determine the minimum concentration of essential oil necessary for effectively inhibiting the growth of the tested bacterium.

**TABLE 7:** Antibacterial activity of *R. vesicarius* methanol extract (80% v/v) using disk-diffusion method*.

Microorganism	ZOI of *R. vesicarius* (1,000 μ g/mL)	ZOI of ampicillin (10 μ g/disk)
*B. subtilis*	13.67 ± 1.5 mm	12.0 ± 1.0 mm
*P. aeruginosa*	11.50 ± 1.0 mm	6.0 ± 0.01 mm

*Test repeated three times, data expressed as mean ± Std. Er., ZOI, Zone of inhibition, mm, millimeter.

**FIGURE 7:**
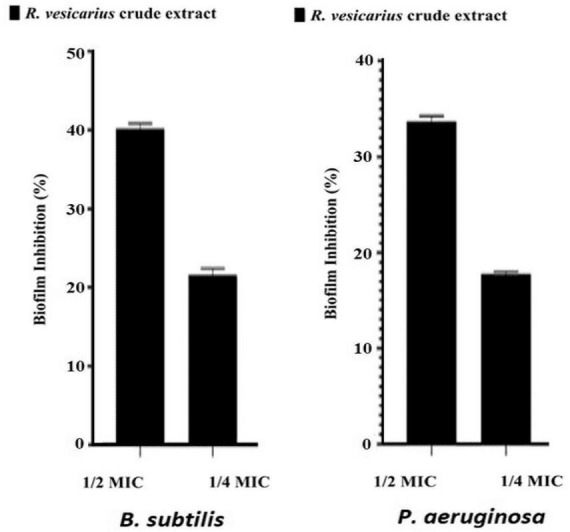
Antibiofilm potential of *R. vesicarius* methanol extract against *B. subtilis* and *P. aeruginosa*. Values are represented as the mean ± SD of three independent experiments.

Additionally, the MBC was conducted to establish the essential oil concentration required to completely eradicate a specific bacterium. The results of these assays and the MBC/MIC ratios, are presented in Table 8. As depicted in the table, variations in MIC and MBC values are observed when assessed across the two different bacterial strains, thereby supporting the findings of the disk-diffusion test. Notably, the Gram-positive strain (*B. subtilis*) demonstrated lower MIC values than its Gram-negative counterparts (*P. aeruginosa*), indicating that the Gram-positives are more susceptible to the plant extract. The MBC of the extract against both bacteria, *B. subtilis* and *P. aeruginosa* is identical at 500 μg/mL, indicating that this concentration of the tested substance is equally effective in completely eradicating both bacterial strains. This emphasizes the possible broad-spectrum potency of the antibacterial principles of *R. vesicarius*. The outcomes of the antibiofilm assessment using a crystal violet assay at sub-MIC concentrations for *R. vesicarius* methanol extract against *B. subtilis* and *P. aeruginosa* are illustrated in Figure 7. The crude extract demonstrated an effective inhibition of biofilm formation in a dose-dependent manner. Specifically, at sub-MIC concentrations, the inhibition of biofilm formation was observed to be 40.19% (1/2 MIC) and 21.60% (1/4 MIC) for *B. subtilis*, and 33.69% (1/2 MIC) and 17.77% (1/4 MIC) for *P. aeruginosa*. Limited information is available on the antibiofilm properties of *R. vesicarius*.

**TABLE 8:** The MIC, MBC, and MBC/MIC ratios of *R. vesicarius* methanol extract (80% v/v) against the chosen bacteria.

Bacterial strain	MIC (μ g/mL)	MBC (μ g/mL)	MBC/MIC
*B. subtilis*	250	500	2
*P. aeruginosa*	500	500	1

MIC, minimum inhibitory concentration, MBC, minimum bactericidal concentration.

## Discussion

The current investigations revealed various phytochemical constituents of biological importance (Table 2). Phytochemical substances refer to secondary metabolic products synthesized by plant cells to perform specific actions crucial for their continued existence in the surrounding ecosystem. Additionally, these substances enhance the plant’s ability to withstand biotic and abiotic challenges (Costa et al., 2023). These compounds exhibit various functional effects on either or indirectly on eukaryotic cells, prokaryotic cells, and viruses. However, they do not play a role in the growth or development of vegetation (Lin et al., 2023). For instance, it has been postulated that polyphenols and flavonoids may mitigate inflammation by impeding the synthesis of pro-inflammatory molecules.

Moreover, polyphenols possess antibacterial properties. Given that numerous pathogenic microorganisms have acquired the capacity to withstand existing antimicrobial drugs, eliminating these germs could potentially be facilitated by utilizing the antibacterial properties of phytochemical molecules (Lin et al., 2023). Furthermore, a prior study aligns in part with our findings. This study reported qualitative phytochemical screening, indicating the presence of flavonoids, saponins, alkaloids, and tannins in 10 to 30-day-old seedlings of *R. vesicarius* (El-Bakry et al., 2011).

The article identified variations in the presence and quantity of specific biologically active constituents in different germination periods (El-Bakry et al., 2011). The Gas Chromatography-Mass Spectrometry (GC-MS) results are represented in Figure 2 and Tables 3, 4. The integration of gas chromatography with mass spectroscopy is an indispensable technique in the analysis of chemical compounds. It is recognized as one of the most widely employed techniques for separating natural products derived from plants (Lalitha et al., 2021).

Mass spectrometry enables the identification of compounds, serving as a valuable source of qualitative data regarding the chemical constituents (Urban, 2016). Based on our research results, we identified sixteen constituents, among which molecular docking analysis indicated inhibition targeting the filamentous temperature-sensitive protein Z (FtsZ), a pivotal component in bacterial cell division. Molecular docking was performed using AutoDock Vina software. Notably, six of these compounds displayed notable efficiency in active site interaction, as evidenced by their low binding scores ranging from −8.3 to −5.0 kcal/mol. This pivotal role of FtsZ in cell division has made it an appealing target for antibiotic medication research. In addition to the GTP binding site, FtsZ features a distinct inter-domain cleft and T7-loop that serve as inhibitory active pockets (Haydon et al., 2008). The FtsZ-inhibitor PC190723 has been used for docking accuracy as reference material (Haydon et al., 2008). PC190723 was docked to the GTP pocket with a score binding energy ranging from −6.3 to 7.9 Kcal/mol and a binding percent 89. Figure 5 shows the 2D and 3D interactions between PC190723 and the active site of the FtsZ protein. The docked poses a high degree of hydrogen bond formation capability. Six conformers make eight bonds with the ARG 143 residue out of the seven conformers docked to the active pocket. The residues VAL 19, THR 103, GLU 139, and ASP 187 were found to be important in hydrogen formation as acceptors. This finding may highlight the importance of the ARG 143 residue in inhibiting the FtsZ protein. Except for Phytol, which did not dock to the FtsZ active site, all of our hits interacted with GTP pockets. The full results are tabulated in Table 4 after the candidates and conformers were ignored, and a score binding energy was lower than 5.0 Kcal/mol. The best hits superimposed with the PC190723 reference are shown in Figure 4. D-amygdalin has demonstrated potential activity superior to PC190723 in terms of binding energy, binding percent, and hydrogen bond formation power. According to an analysis of the literature, the lactic acid bacteria strains separated from fermented rice noodles and containing D-amygdalin exhibited high inhibitory action against *Helicobacter pylori* (Techo et al., 2019). Also, amygdalin has stronger anti-HepG2 activity (Zhou et al., 2012). The best conformer of D-amygdalin that interacted with the FtsZ GPT site is shown in Figure 6. The significant hits involved in the extracts of *R. vesicarius*’s inhibitory action against *B. subtilis* and *P. aeruginosa* have been revealed by this docking data.

Our investigation also revealed the presence of 15 elements, as detected by elemental analysis, arranged in the following order: K > Ca > Cl > Al > Si > Fe > P > S > Mg > Ti > Sr > Zn > Mn > Br > Cr. While not required by all plant species, these elements have been reported to be essential in promoting plant growth and improving resilience to biotic stresses, such as pathogens and herbivory. Furthermore, they have demonstrated the ability to alleviate abiotic stresses, including salinity, drought, and nutrient toxicity or deficiency (Pilon-Smits et al., 2009; Pandey, 2015). Interestingly, our results showed that toxic heavy metals such as Cd and Pb were not found in the *R. vesicarius* sample from the Hail region, indicating that the soil there is pure and unpolluted. In recent years, heavy metals such as Cd, Co, Pb, and Cu in agricultural soil and cultivated medicinal plants due to the accumulation of residues and pollutants has become an issue of interest and research. This is particularly concerning as medicinal plants are freely sold in open-air markets without chemical or biological analysis (Sarma et al., 2011; Shen et al., 2017). Our research aligns with a prior investigation that identified *R. vesicarius* as a valuable reservoir of essential and health-promoting minerals, including but not limited to calcium, copper, iron, magnesium, potassium, sodium, and zinc (Alfawaz, 2006).

In our investigation, antioxidant evaluation unveiled notable antioxidant attributes at reduced concentrations when juxtaposed with those of ascorbic acid, butylated hydroxytoluene, and β-carotene. Our results are consistent with a prior investigation that demonstrated the antioxidant activity of the methanolic extract of *R. vesicarius* (Lobo et al., 2010; Khan et al., 2014). Antioxidants neutralize harmful free radicals. Free radicals emerge as natural byproducts of the body’s regular metabolic activities within biological systems. Antioxidants play a crucial role in safeguarding against various ailments by neutralizing these free radicals (Lobo et al., 2010; Snoussi et al., 2023).

The antioxidant potential of *R. vesicarius* was assessed using parameters such as total phenols, total tannins, flavonoids, DPPH scavenging activity, beta-carotene, and ABTS IC_50_ (mg/mL). We compared these measurements to established standard molecules; the results are represented in Table 4. Upon comparing the results with those of ascorbic acid (AA), butylated hydroxytoluene (BHT), and β-carotene, it is evident that *R. vesicarius* exhibits promising antioxidant properties at lower concentrations. It was published that the DPPH scavenging activity test revealed that *R. vesicarius* extract derived from leaves (at the early vegetative stage) exhibited the lowest IC_50_ value, signifying the highest effectiveness compared to the other plant parts, with an IC_50_ value of 0.345 ± 0.005 mg/mL. It varies with the vegetative growth stage (El-Bakry et al., 2012). Moreover, the presence of an antioxidant significantly reduced the stability of the DPPH free radical, as evidenced by strong and visible UV-Vis absorption at 517 nm.

The decrease in the ability of methanol solution to absorb the DPPH radical at 517 nm correlated with increased antioxidant activity. Experimentally determined sample concentrations resulting in a 50% inhibition (IC_50_) of DPPH radical scavenging activity were compared to evaluate both DPPH scavenging action and the efficiency of extraction solvents. A lower IC_50_ value denotes higher antioxidant activity (Brighente et al., 2007). Our study also revealed the presence of tannins, flavonoids, and total phenolic content in *R. vesicarius* extract. Different extracts demonstrated the ability to quench stable ABTS and O_2_ free radicals, allowing for the assessment of their free radical scavenging capacity. However, the extracts exhibited significantly lower capacities to neutralize O_2_ and ABTS radicals compared to standard compounds (Schinella et al., 2010). Polyphenols, encompassing phenolic acids, tannins, coumarins, anthraquinones, and flavonoids, were identified as significant antioxidants isolated from higher plants. Their redox properties enable them to act as reducing agents, singlet oxygen quenchers, hydrogen donors, and potential metal ion chelators (Noumi et al., 2023).

Utilizing the Folin–Ciocalteu phenol reagent, the total polyphenol content of *R. vesicarius* was quantified at 3.72 ± 0.05 mg gallic acid equivalent per gram of dry plant weight. Finally, the development of an innovative drug, such as an antibiotic, must incorporate an antioxidant agent to mitigate side effects, as suggested by insights from gentamicin, a potent agent targeting Gram-negative bacteria. However, it leads to nephrotoxicity as a significant complication associated with gentamicin administration, attributed to oxygen metabolites and reactive oxygen species (ROS) (Al-Amer et al., 2023).

Phytomedicines offer potent therapeutic effects due to their various endogenous antioxidant compounds. These include phenolics, tannins, flavones, flavonols, flavonoids, alkaloids, saponins, terpenoids, and reducing Sugars. Investigation of medicinal plants is a subject of great interest because botanicals are known to contain compound active antioxidant substances. This concern has awakened curiosity around the globe for a medicinal plant as a credible supplier of potent antioxidants.

The antioxidant activities of *R. vesicarius* extract can be associated to the presence of many metabolites like n-Hexadecanoic acid (Kalpana et al., 2012), Phytol (Pejin et al., 2014), methyl stearidonic (Jitendra et al., 2015), Methyl isohexadecanoate (Mohamed et al., 2018), linoleic acid methyl ester (Gıdık, 2021), and amygdalin (Barakat et al., 2022). In fact, previous researchers have reported that n-Hexadecanoic acid has antioxidant activity as follows: DPPH (30.19–89.13% at 100–500 μg/ml), ABTS (42.18–83.86% at 100–500 μg/ml), reducing power (0.02–0.16% at 100–500 μg/ml), nitric oxide (18.65–73.17% at 100–500 μg/ml), and superoxide (17.18–81.21% at 100–500 μg/ml), respectively (Kalpana et al., 2012). It has been reported that the diterpene member (Phytol) reduced the production of many radicals by 56, 50, and 48% for carbon-dioxide anion radical, methoxy radical, and DPPH radicals, respectively (Pejin et al., 2014).

On the other side, our results indicated that *in vitro* antibacterial analysis displayed inhibitory effects against *B. subtilis* MTCC121 and *P. aeruginosa* MTCC 741, with inhibition zones measuring 13.67 ± 1.0 mm and 11.50 ± 1.0 mm, respectively. The MIC and MBC values ranged from 250 to 500 μg/mL. Furthermore, *R. vesicarius* demonstrated antibiofilm activity. Our claim finds support in existing literature, exemplified by a study indicating the potent antibacterial activities of various alcoholic extracts of *R. vesicarius* against *Pseudomonas aeruginosa*, *Escherichia coli*, *Klebsiella pneumoniae*, *Streptococcus pyogenes*, and *Staphylococcus aureus*. Furthermore, numerous additional studies corroborate our findings and validate the presence of antibacterial agents in *R. vesicarius* (El-Bakry et al., 2012; Shah et al., 2015; Chaity et al., 2020). Our findings were relatively in concordance with a previously published article, which reported that for different bacterial strains, the highest MIC value of *R. vesicarius* was 250 mg/mL, and the lowest MIC value was 31.25 mg/mL (Tukappa and Londonkar, 2013). Furthermore, the MBC/MIC ratio was computed as detailed in Table 6. Our observations reveal a range of 2.0 to 1.0 for this ratio across the examined bacterial strains, underscoring the evident bactericidal efficacy of the methanol extract derived from *R. vesicarius* against these bacterial species. The classification of a tested compound as possessing bactericidal properties occurs when the MBC/MIC ratio is at or below 4.0, while a ratio exceeding 4.0 indicates the compound exhibits bacteriostatic attributes (Abdallah, 2016). The assessment of the MBC/MIC ratio serves as a critical parameter for evaluating a substance’s ability to eliminate bacteria. The primary objective in determining the MBC/MIC ratio was to enhance the understanding of the mode of action associated with the tested extract (Jeddi et al., 2023). It was stated that the MBC/MIC ratios of various *R. vesicarius* extracts against three bacterial strains were less than 4, indicating that all *R. vesicarius* extracts exhibit bactericidal properties (Tukappa and Londonkar, 2013). Further investigations are recommended to comprehensively elucidate the growth kinetics and mode of action of microorganisms when exposed to treatment with *R. vesicarius* extract, both *in vitro* and *in vivo*.

Nevertheless, a published paper indicates that *R. vesicarius* exhibits promise as an antibiofilm agent against various bacteria, including *Staphylococcus epidermidis*, *Staphylococcus aureus*, *Proteus vulgaris*, *Pseudomonas aeruginosa*, and *Klebsiella pneumoniae*. Moreover, it has demonstrated efficacy in addressing infections associated with indwelling medical devices (Fady et al., 2023). Another study demonstrated that the anti-biofilm attribute of another species within the genus *Rumex*, *Rumex dentatus*, is directly proportional to the concentration of extracts observed at both 48 and 72 h (Najafabadi et al., 2020). Biofilms are believed to be linked to 80% of microbial infections, and it is widely acknowledged that the growth of microorganisms within biofilms can significantly bolster their resistance to antimicrobial agents. Consequently, conventional antimicrobial therapies frequently prove ineffective in eradicating biofilms from infection sites. This underscores the imperative need for innovative anti-biofilm agents that target novel mechanisms and exhibit unique modes of action (Brackman and Coenye, 2015; Akova et al., 2021).

## Conclusion

In conclusion, the investigation of *R. vesicarius* from the Hail highlands in Saudi Arabia revealed a diverse array of phytochemical constituents, identifying 16 compounds. Docking was performed between the identified organic compounds and the filamentous temperature-sensitive protein Z (FtsZ), essential for bacterial cell division. Following the formation of single-stranded filaments, FtsZ constructs the exceedingly dynamic Z-ring scaffold. The docking results showed that 6 out of 16 substances inhibited FtsZ, with binding scores ranging from −8.3 to −5.0 kcal/mol. The docked poses a high degree of hydrogen bond formation capability to ARG 143 residue in the active pocket.

Meanwhile, VAL 19, THR 103, GLU 139, and ASP 187 contribute to hydrogen acceptor formation. Elemental analysis revealed the presence of 15 elements, excluding heavy metals, with potassium (K), calcium (Ca), and chlorine (Cl) prevailing—recognized as essential nutrients and health-promoting elements. The antioxidant analysis demonstrated notable properties at lower concentrations than commonly known antioxidants, potentially offering cell protection against free radicals implicated in various chronic diseases. The antibacterial assessment showcased significant inhibitory effects against *Bacillus subtilis* and *P. aeruginosa*, with promising MIC and MBC values. The MBC/MIC ratio suggested potential bactericidal efficacy.

Additionally, the study underscored the antibiofilm formation activity of *R. vesicarius*. While *B. subtilis* may play positive roles in specific food processes, *P. aeruginosa* is generally regarded as harmful and undesirable in food due to its potential pathogenicity and spoilage effects. These findings provide valuable insights into the phytochemical composition and biological activities of the edible plant *R. vesicarius*, supporting its potential application in pharmaceutical and medicinal contexts. However, further research is recommended to elucidate specific mechanisms of action and validate its therapeutic potential through comprehensive *in vitro* and *in vivo* studies. More comprehensive experimental evidence, such as *in vitro* and *in vivo* assays, is recommended for future investigations to enhance the potential impact of biological activity and contribution to the field.

## Data availability statement

The original contributions presented in this study are included in this article/supplementary material, further inquiries can be directed to the corresponding author.

## Author contributions

AMES: Conceptualization, Funding acquisition, Project administration, Supervision, Writing – original draft, Writing – review & editing. EA: Methodology, Resources, Writing – original draft. NA: Conceptualization, Resources, Writing – review & editing. HI: Formal analysis, Supervision, Writing – original draft. MA: Methodology, Validation, Visualization, Writing – original draft. AJ: Methodology, Resources, Software, Writing – original draft. SS: Methodology, Resources, Writing – review & editing. MS: Conceptualization, Writing – original draft.
